# Supplementary material for: Synthesis and Photolytic Assessment of Nitroindolinyl-Caged Calcium Ion Chelators
Source: Molecules. 2022 Apr 20;27(9):2645. doi: 10.3390/molecules27092645 (PMC9104977; doi:10.3390/molecules27092645)
Supplement: Supplementary file 1 [file molecules-27-02645-s001.zip › molecules-1662162-supplementary.pdf]

Supplementary Information for:

Synthesis and Photolytic Assessment of Nitroindolinyl-Caged Calcium Ion Chelators†

George Papageorgiou [1, *] and John E. T. Corrie [2,‡]

[1]Chemical Biology STP, The Francis Crick Institute, 1 Midland Road, London NW1 1AT, UK
[2]MRC National Institute for Medical Research, The Ridgeway, Mill Hill, London NW7 1AA, UK
***Correspondence: George.Papageorgiou@crick.ac.uk**

†This paper is dedicated to the memory of Dr David Ogden, a fine scientist and collaborator, who sadly passed away recently.
‡Retired: The experimental part of this work was carried out at the MRC National Institute for Medical Research, The Ridgeway, Mill Hill, London, NW7 1AA.

Contents:

Figure S1 ^{1}H NMR spectrum of compound **3** S2
Figure S2 ^{1}H NMR spectrum of compound **4** S3
Figure S3 ^{1}H NMR spectrum of compound **5** S4
Figure S4 ^{1}H NMR spectrum of compound **6** S5
Figure S5 ^{1}H NMR spectrum of compound **6a** S6
Figure S6 ^{1}H NMR spectrum of compound **7** S7
Figure S7 ^{1}H NMR spectrum of compound **9** S8
Figure S8 ^{1}H NMR spectrum of compound **10** S9
Figure S9 ^{1}H NMR spectrum of compound **11** S10
Figure S10 ^{1}H NMR spectrum of compound **12** S11
Figure S11 ^{1}H NMR spectrum of compound **13** S12
Figure S12 ^{1}H NMR spectrum of compound **14** S13
Figure S13 ^{1}H NMR spectrum of compound **15** S14
Figure S14 ^{1}H NMR spectrum of compound **16** S15
Figure S15 ^{1}H NMR spectrum of compound **17** S16
Figure S16 1H NMR spectrum of compound **18** S17
Figure S17 ^{1}H NMR spectrum of compound **18a** S18
Figure S18 1H NMR spectrum of compound **19** S19
Figure S19 ^{1}H NMR spectrum of compound **21** S20
Figure S20 ^{1}H NMR spectrum of compound **23** S21
Figure S21 ^{1}H NMR spectrum of compound **24** S22

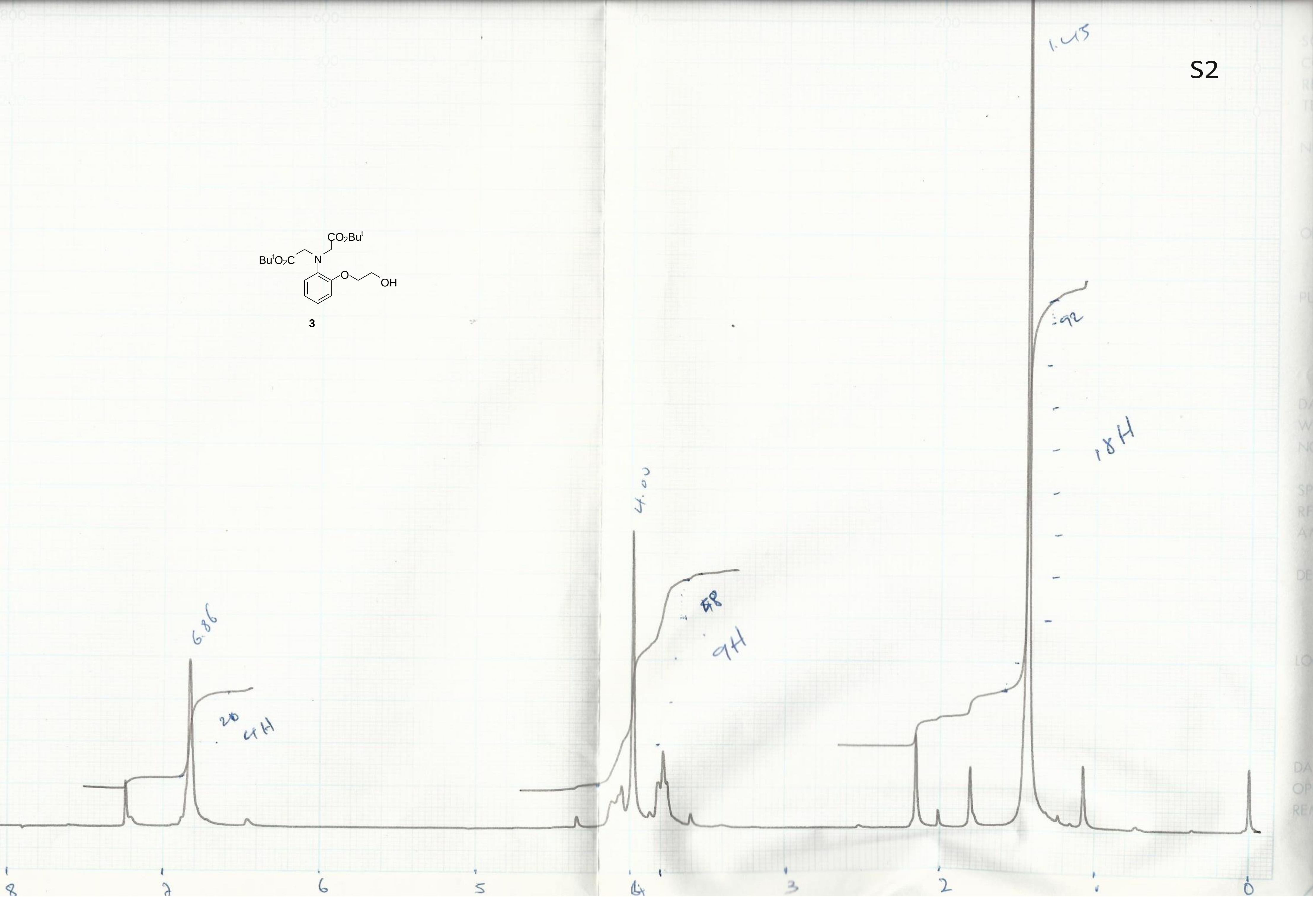

Figure S1 Compound 3 (90 MHz, CDCl3)

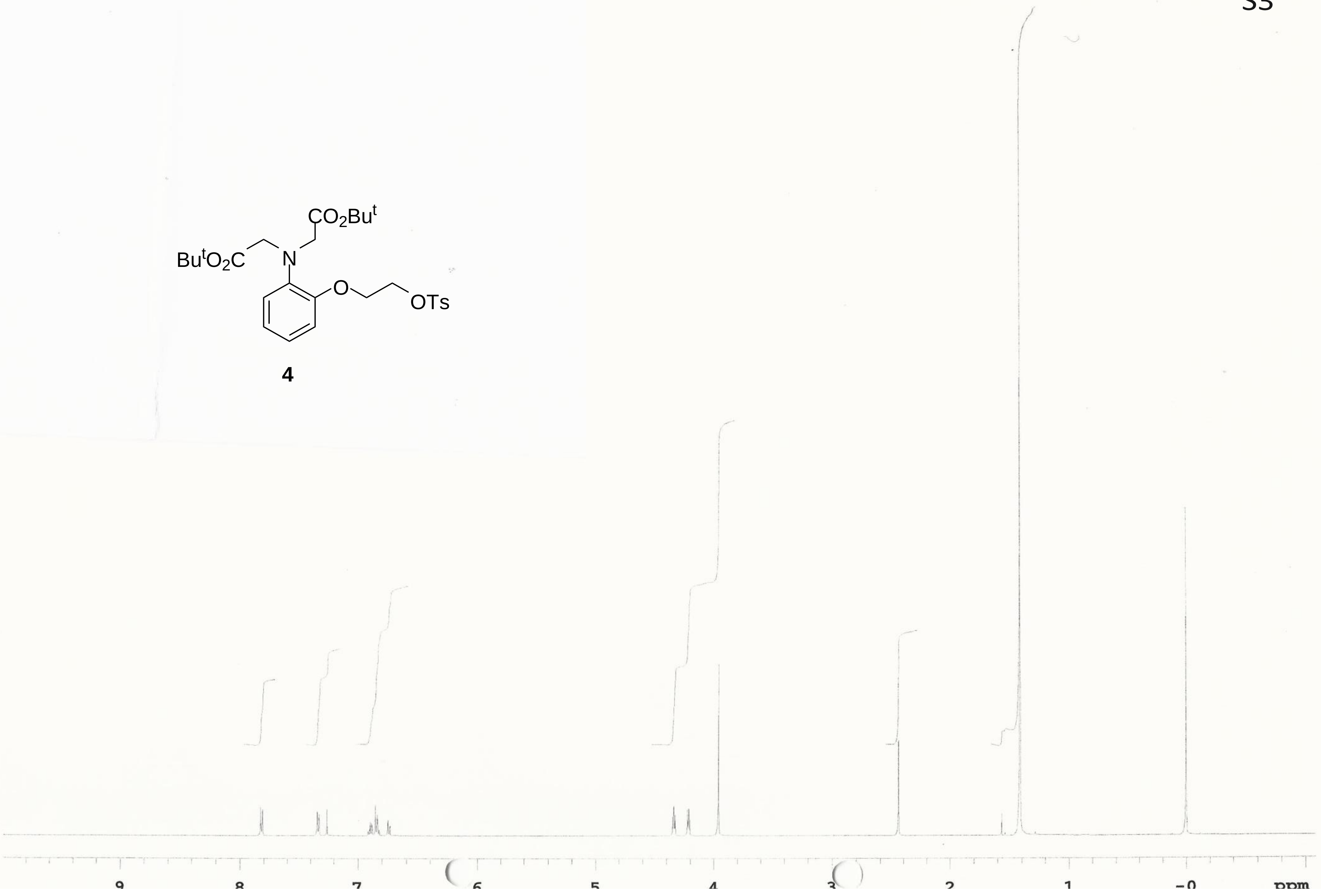

Figure S2 Compound 4 (500 MHz, CDCl3)

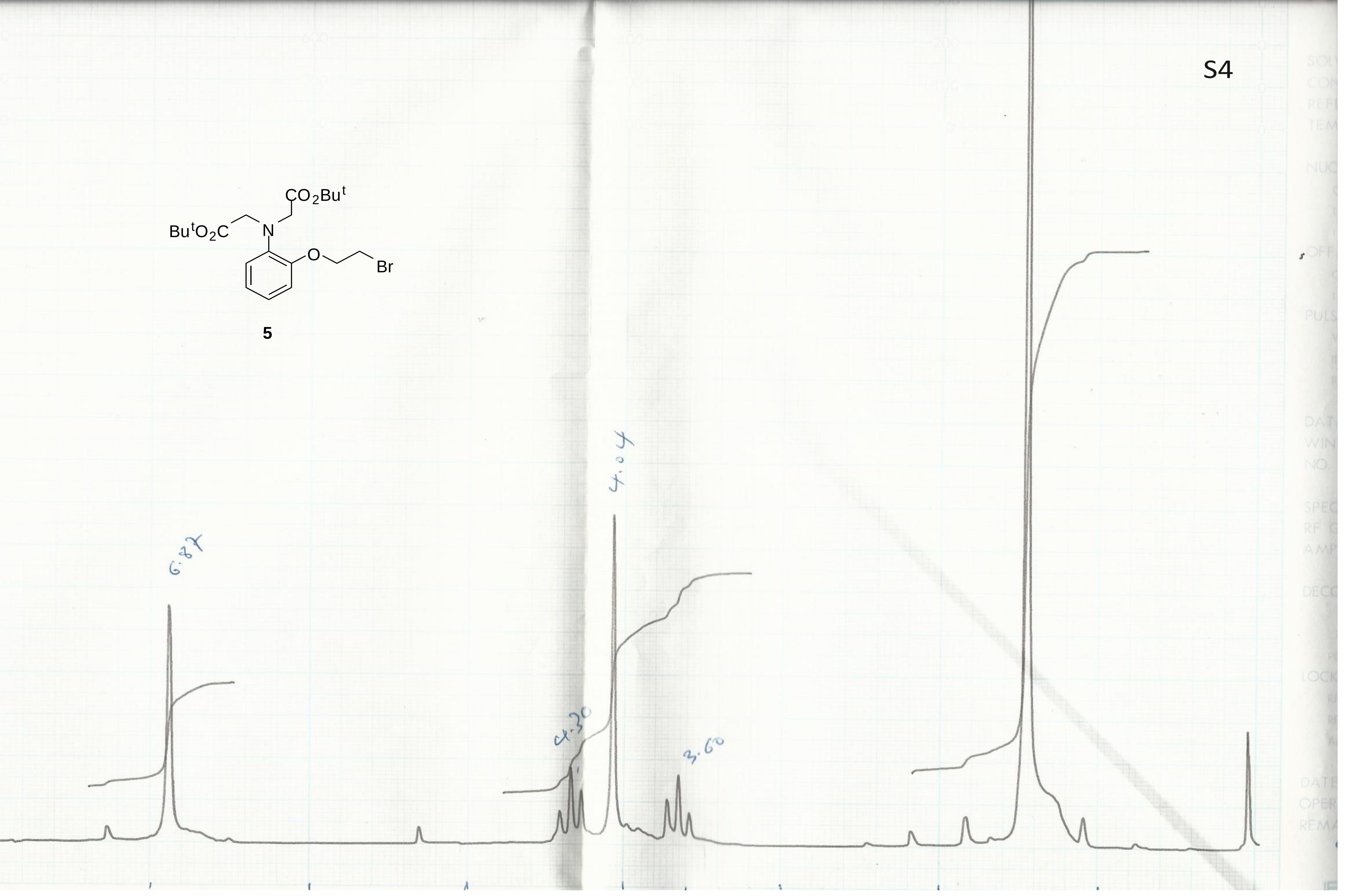

Figure S3 Compound 5 (90 MHz, CDCl3)

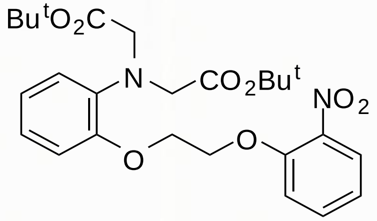

6

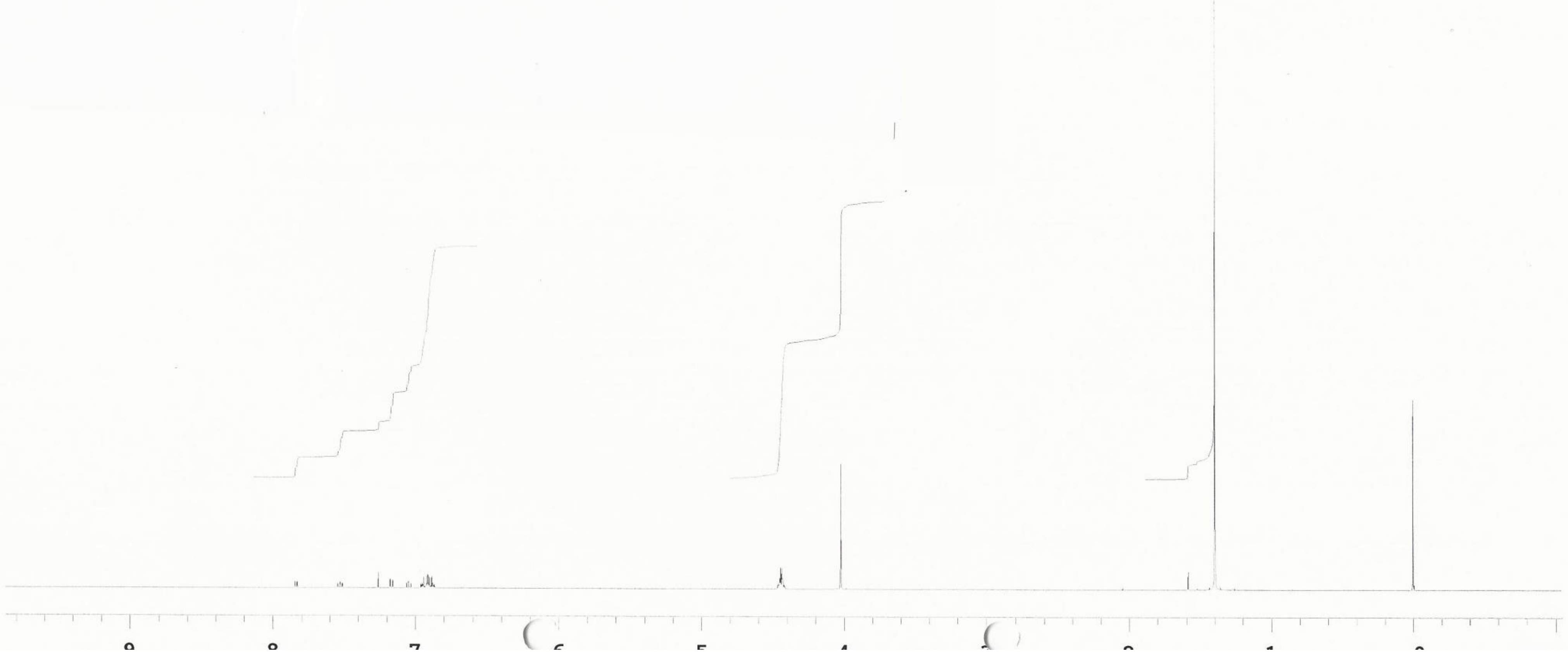

Figure S4 Compound 6 (500 MHz, CDCl3)

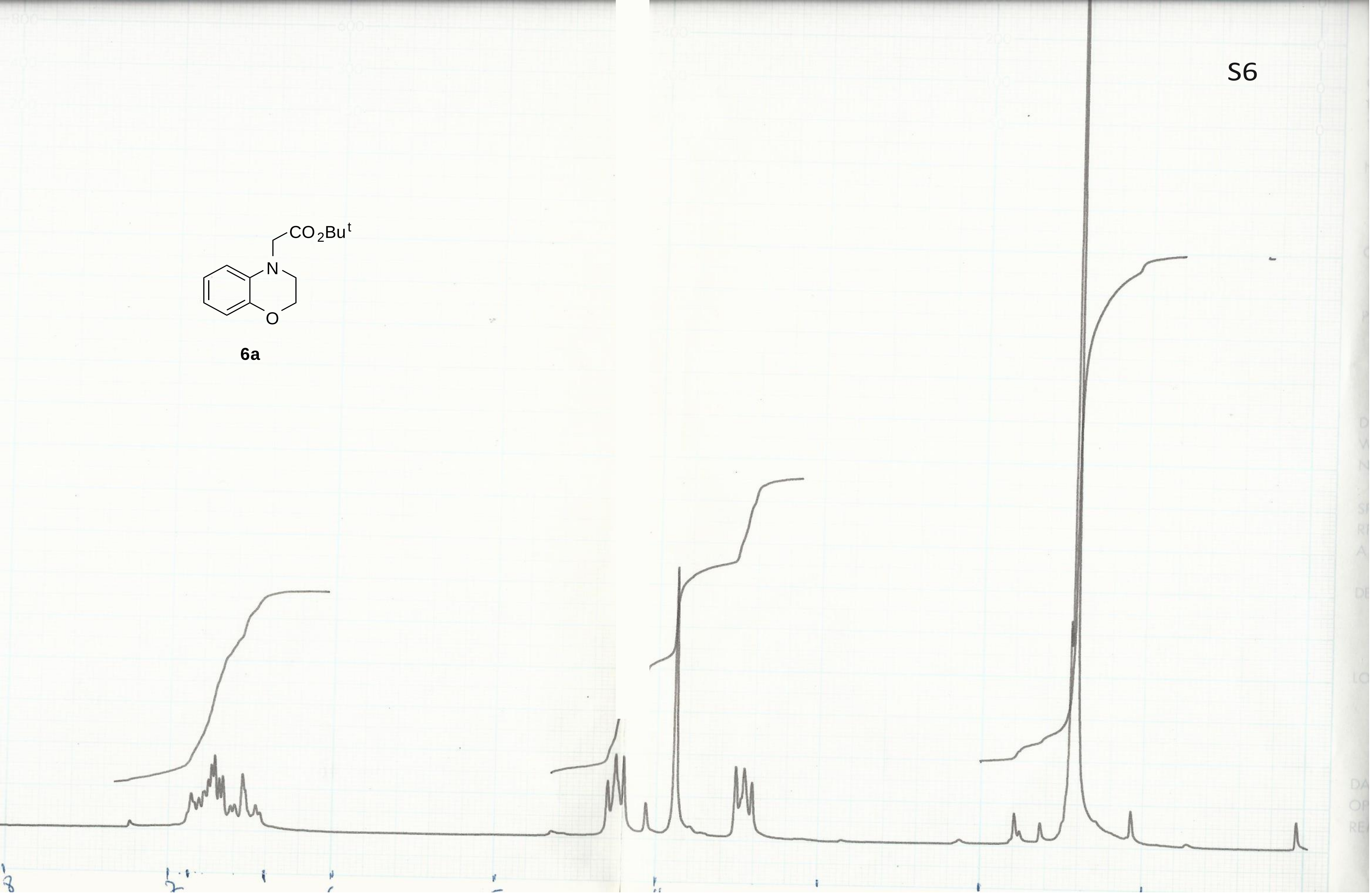

Figure S5 Compound 6a (90 MHz, CDCl3)

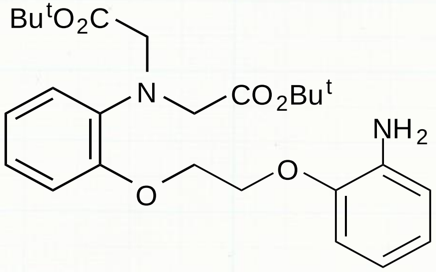

7

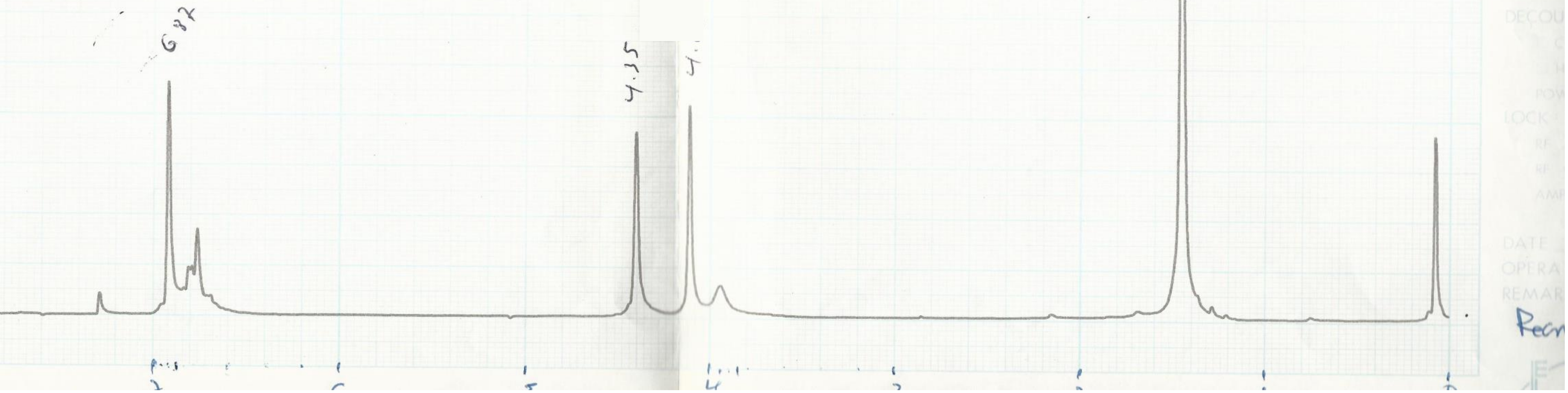

Figure S6 Compound 7 (90 MHz, CDCl3)

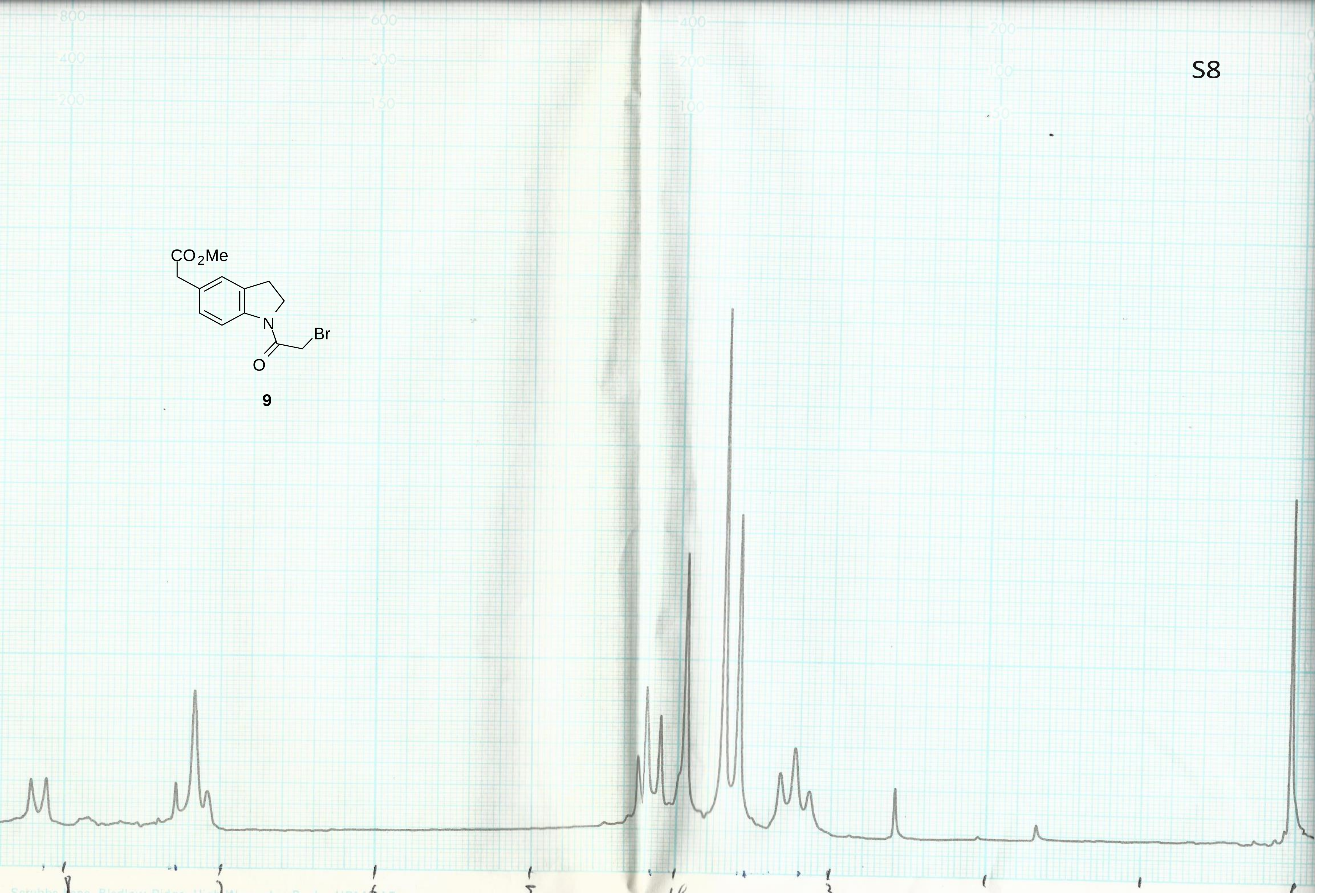

Figure S7 Compound 9 (90 MHz, CDCl3)

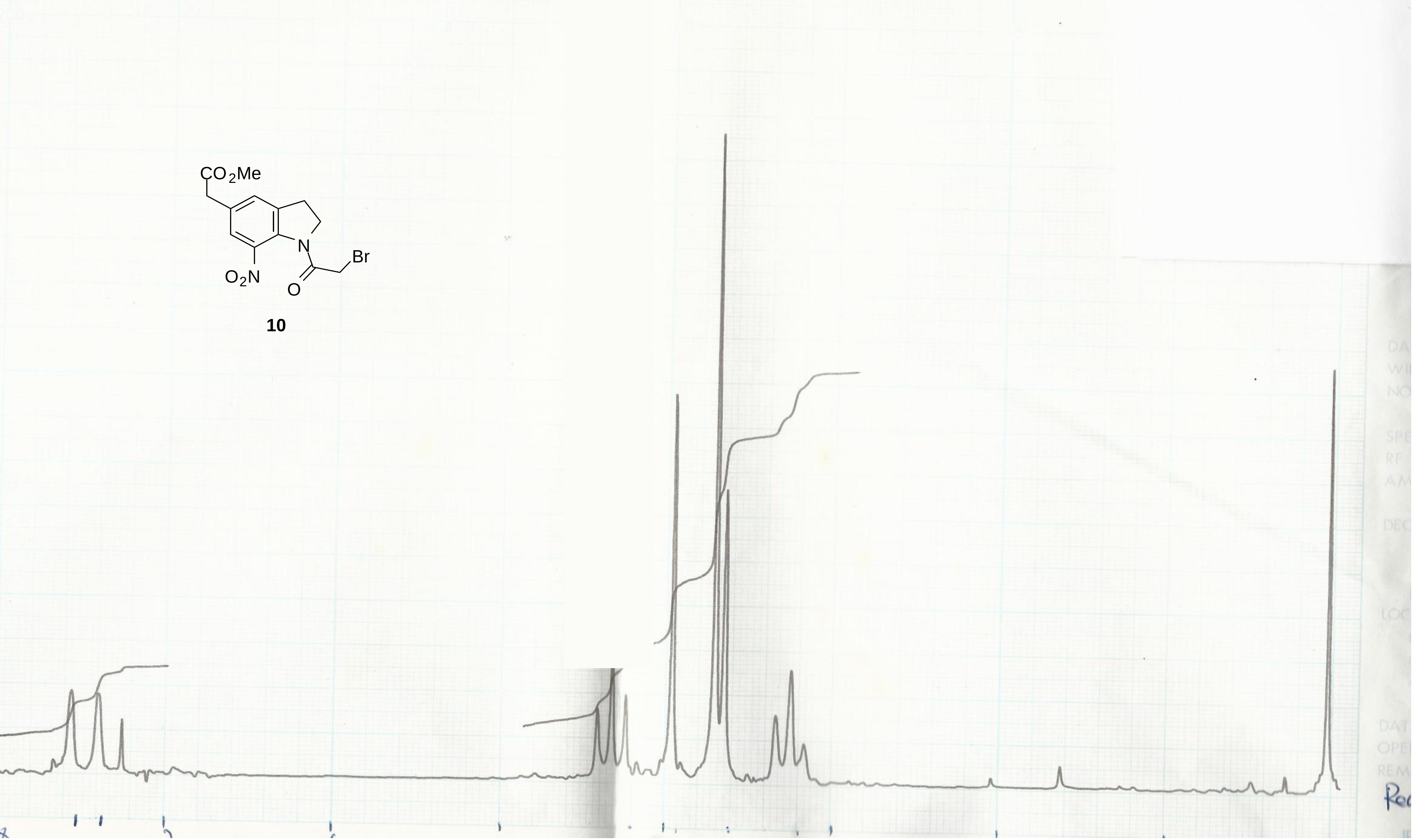

Figure S8 Compound 10 (90 MHz, CDCl3)

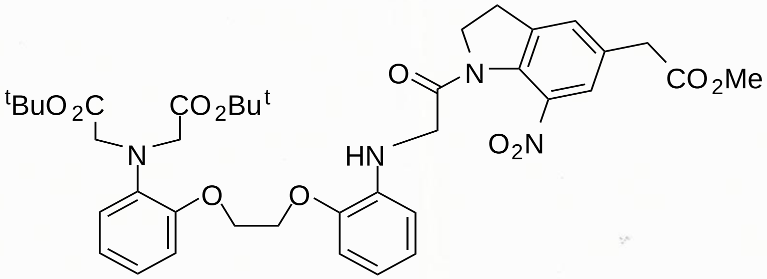

11

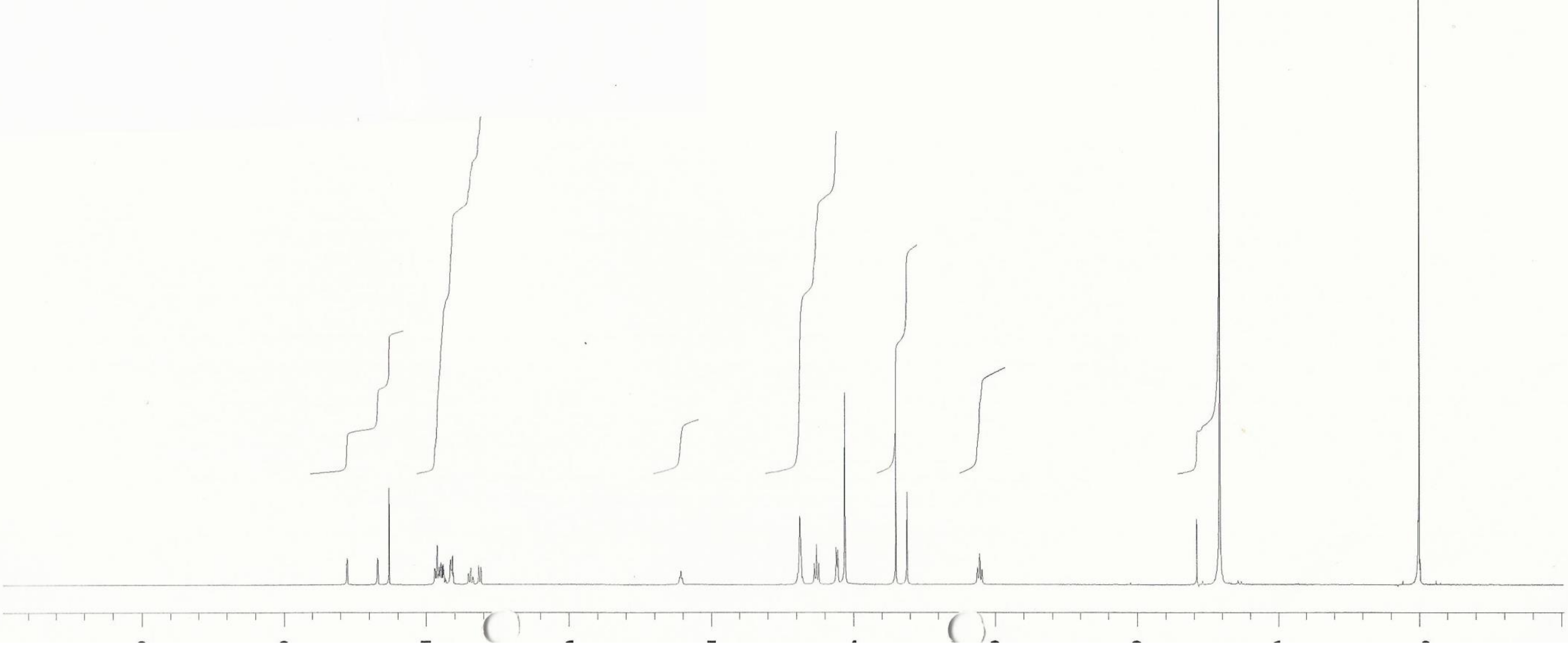

Figure S9 Compound 11 (500 MHz, CDCl3)

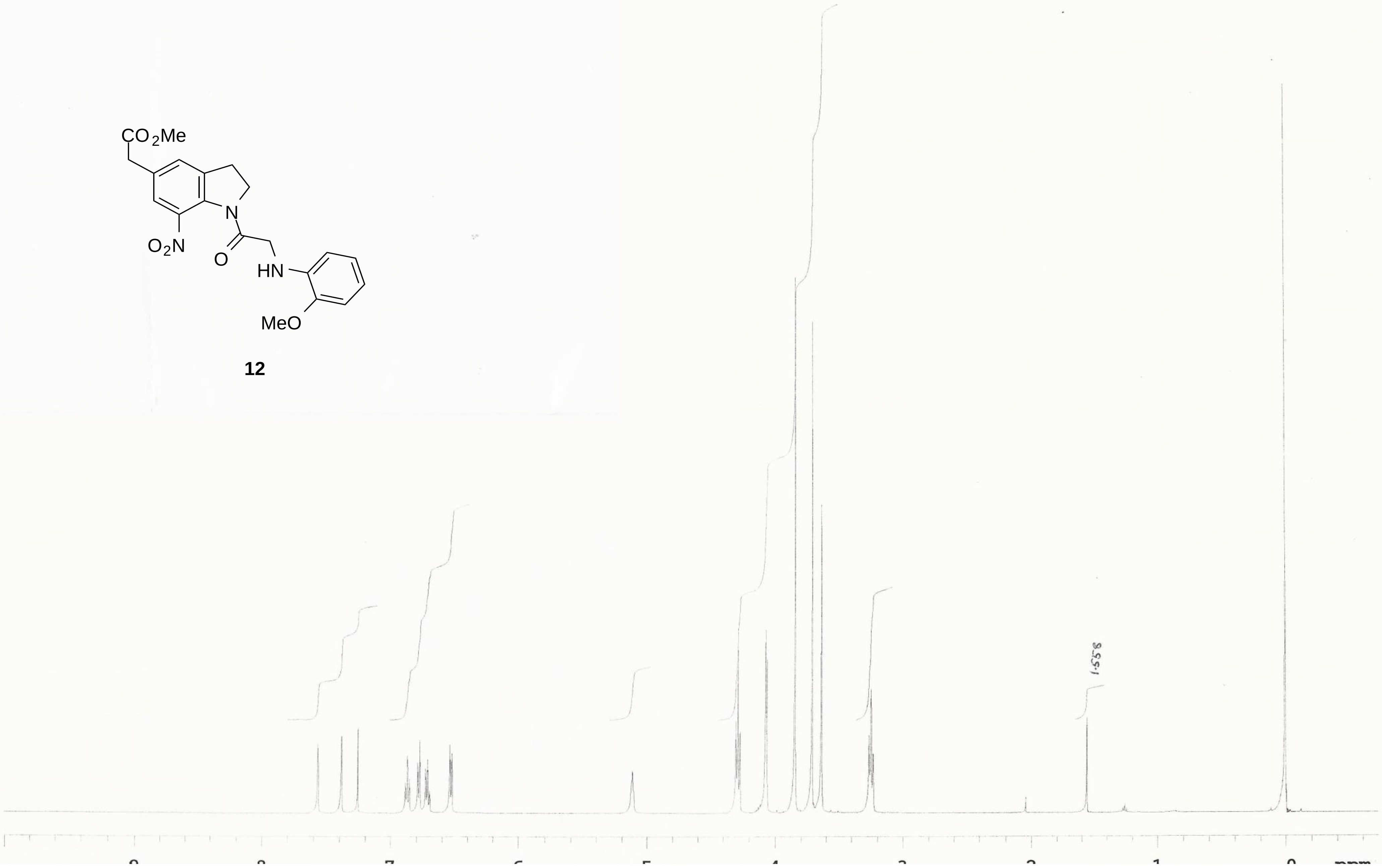

Figure S10 Compound 12 (500 MHz, CDCl3)

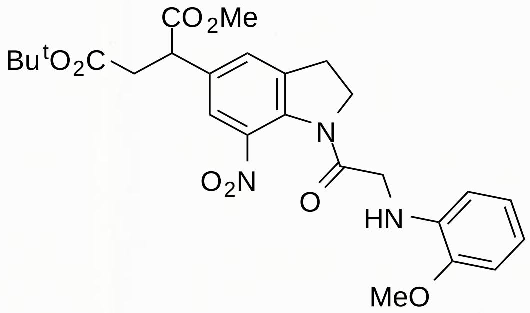

13

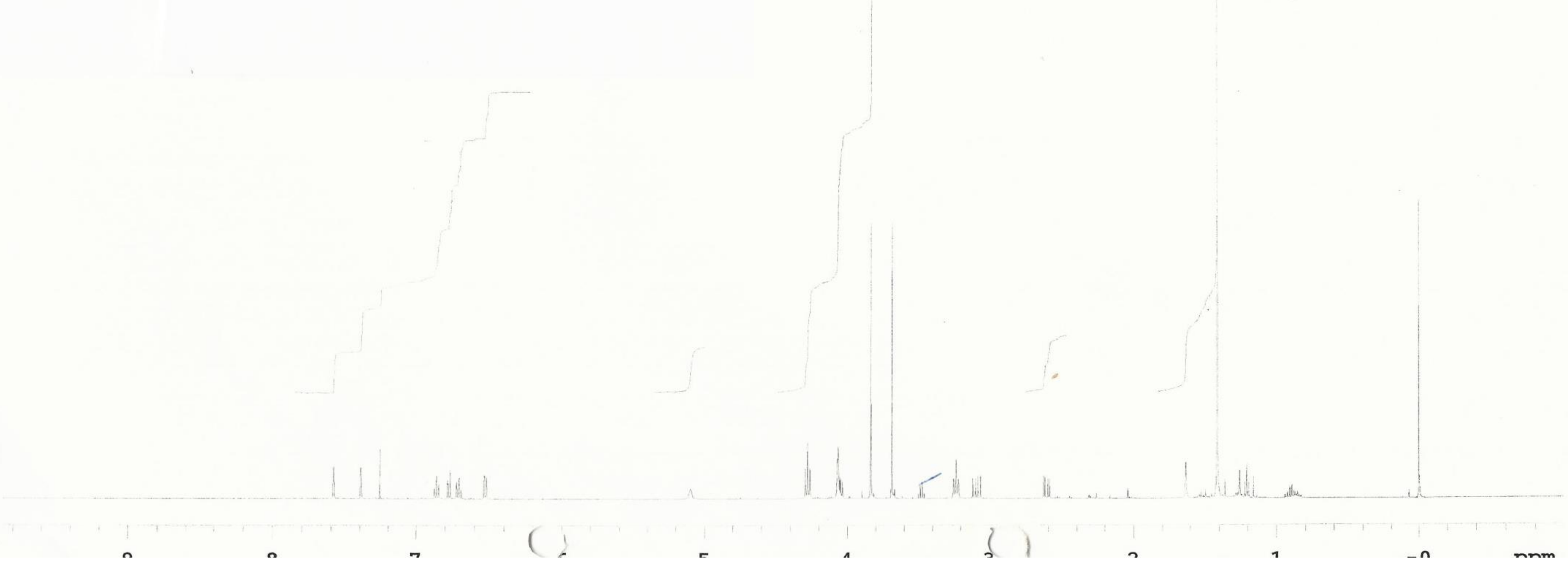

Figure S11 Compound 13 (500 MHz, CDCl3)

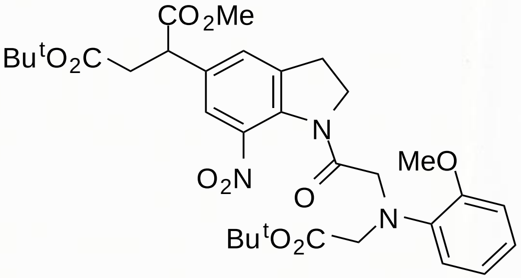

14

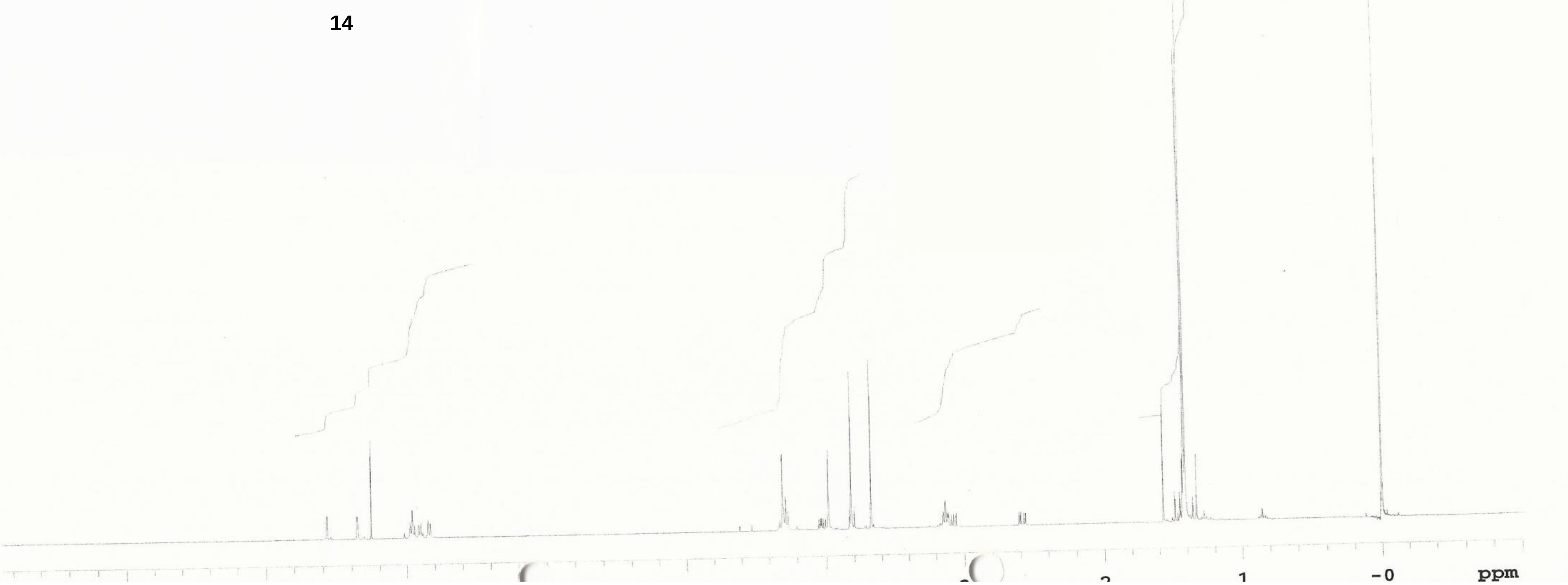

Figure 12 Compound 14 (500 MHz, CDCl3)

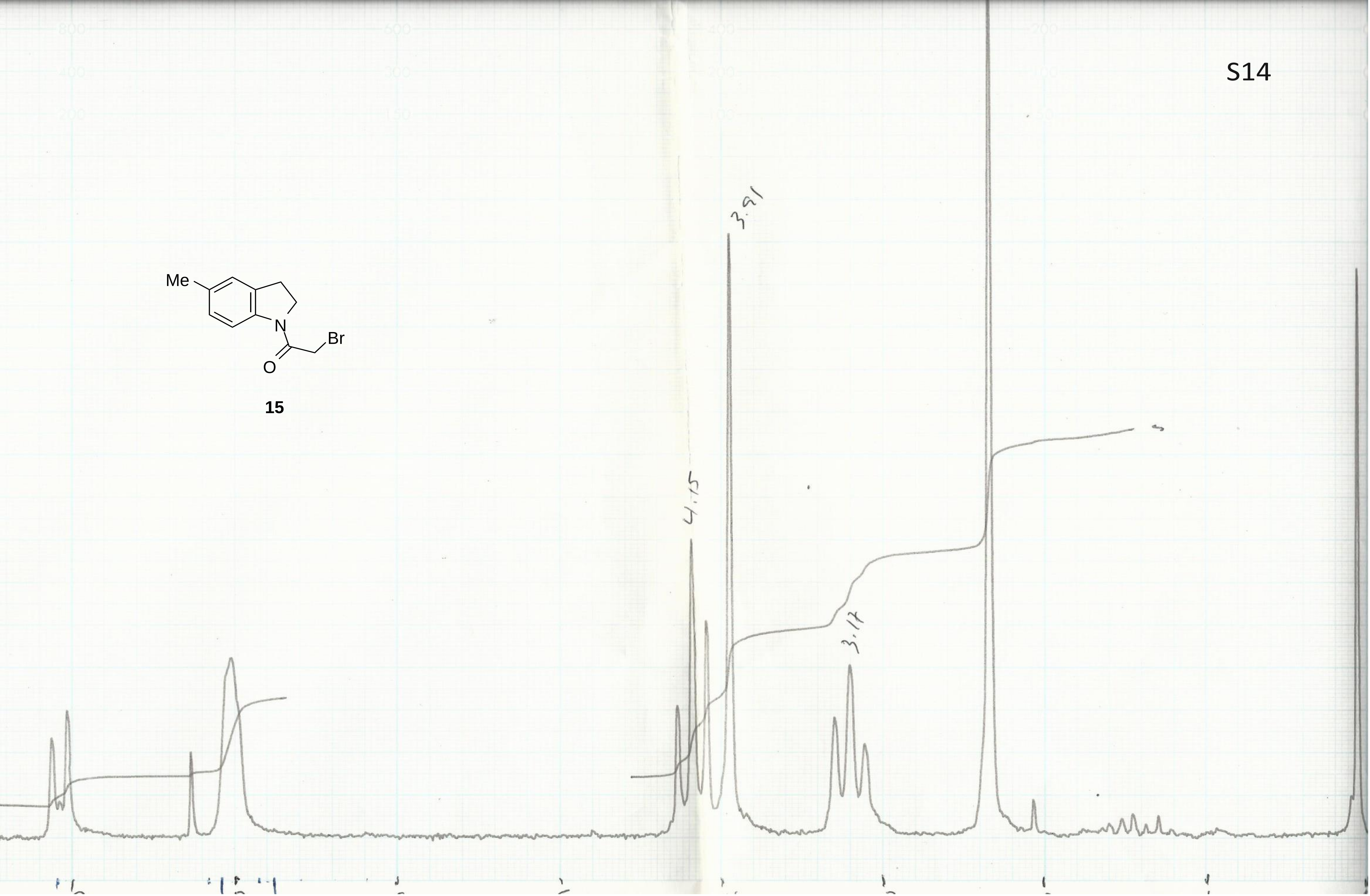

Figure S13 Compound 15 (90 MHz, CDCl3)

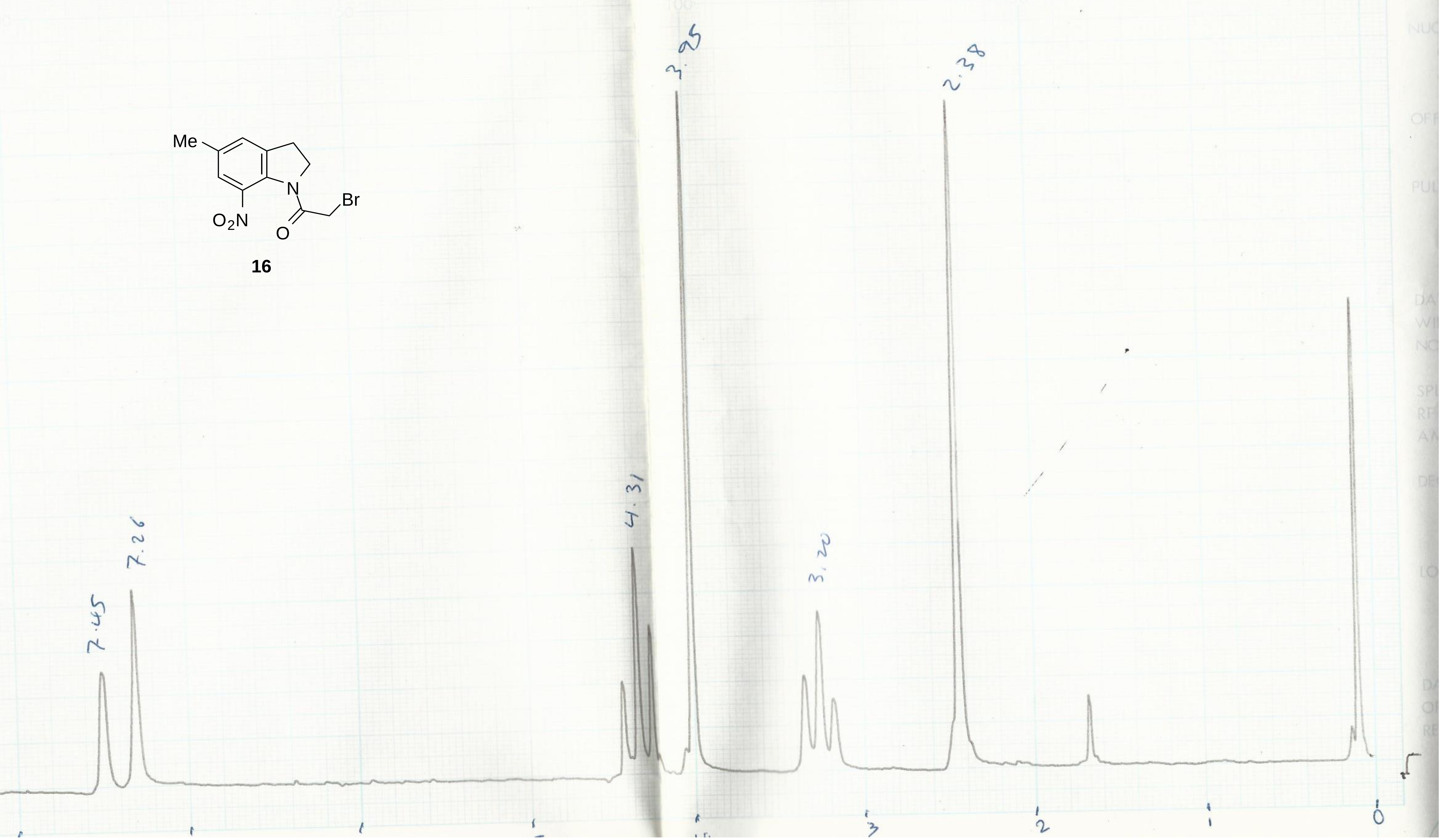

Figure S14 Compound 16 (90 MHz, CDCl3)

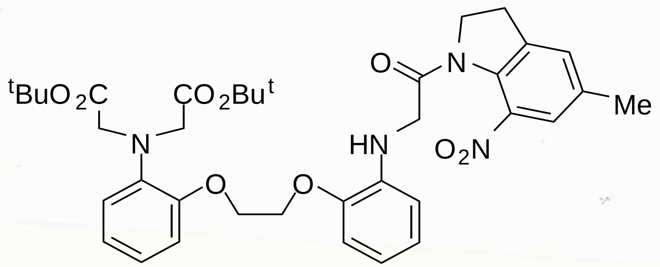

17

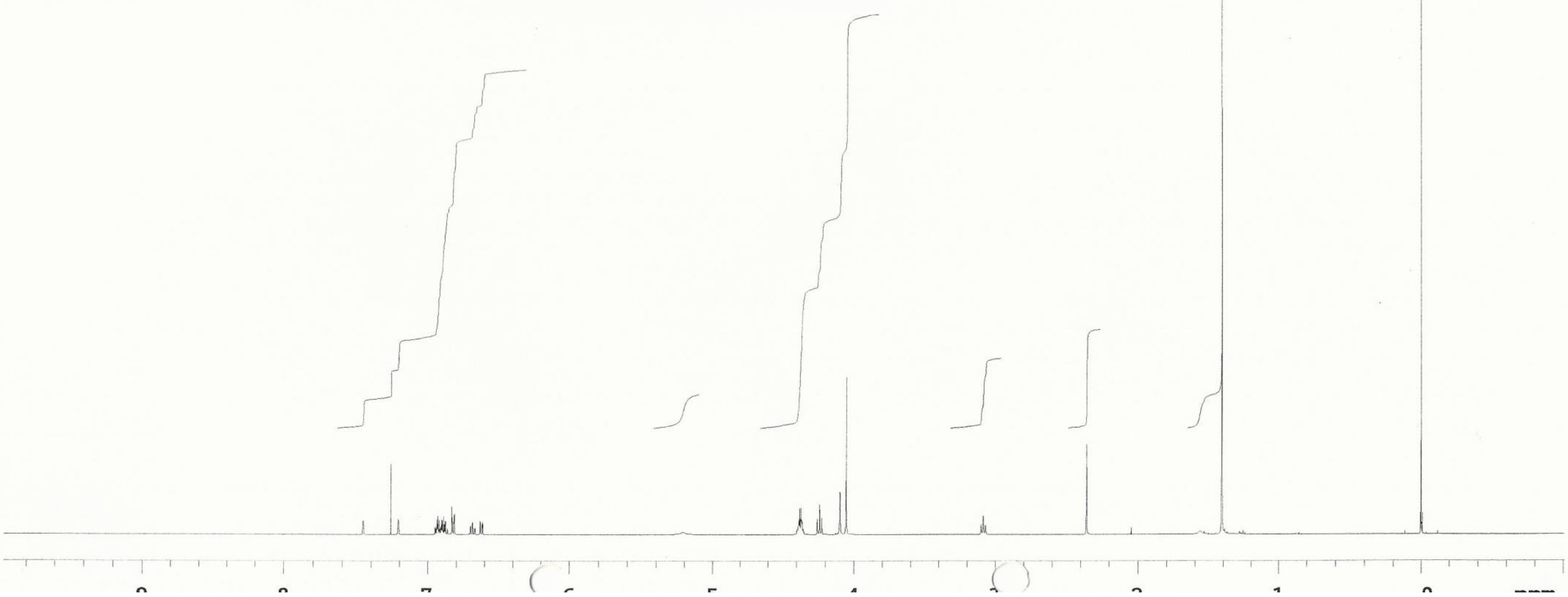

Figure S15 Compound 17 (500 MHz, CDCl3)

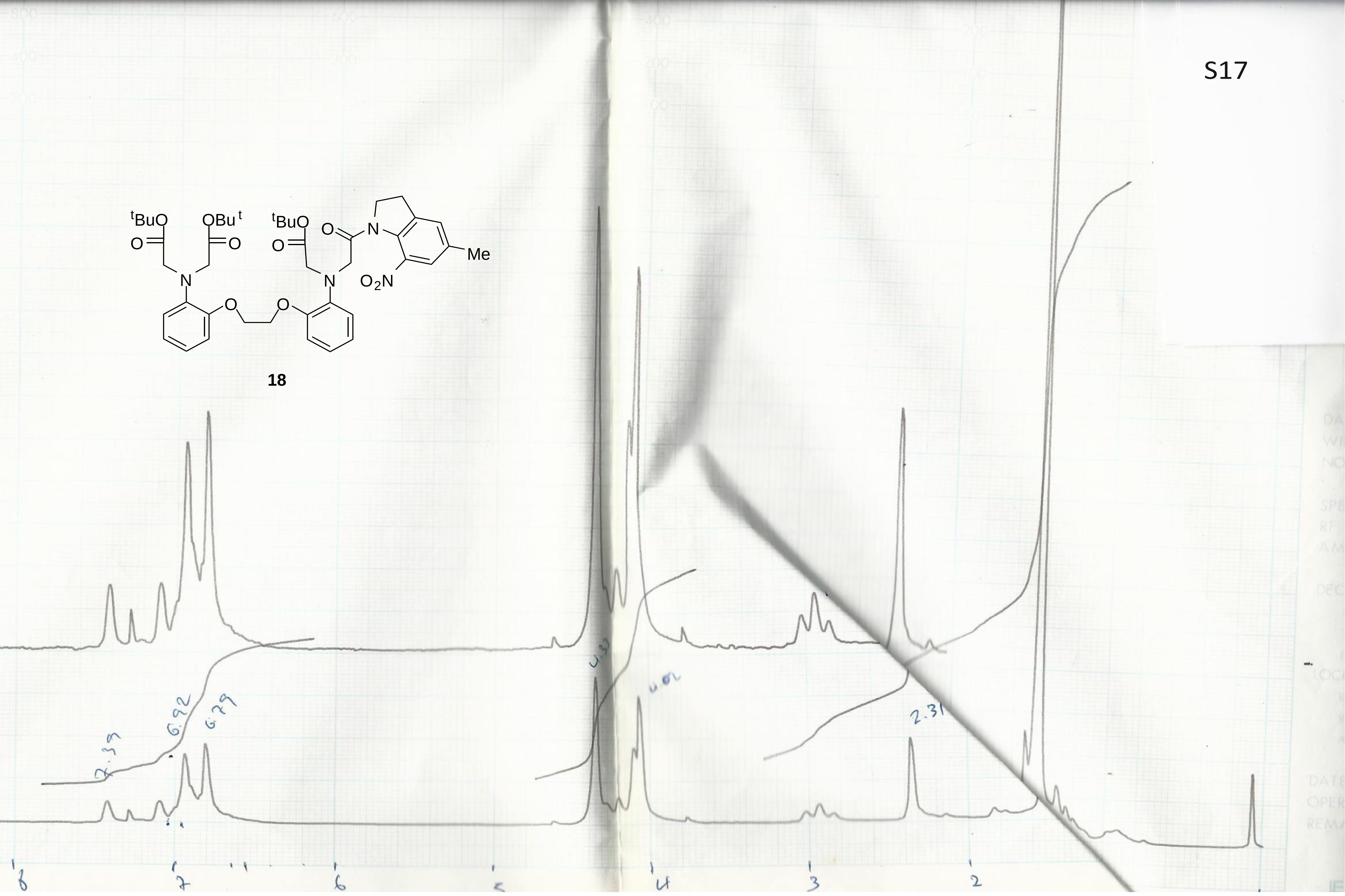

Figure S16 Compound 18 (90 MHz, CDCl3)

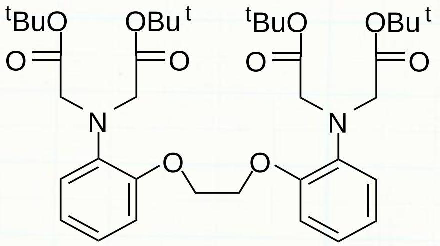

18a

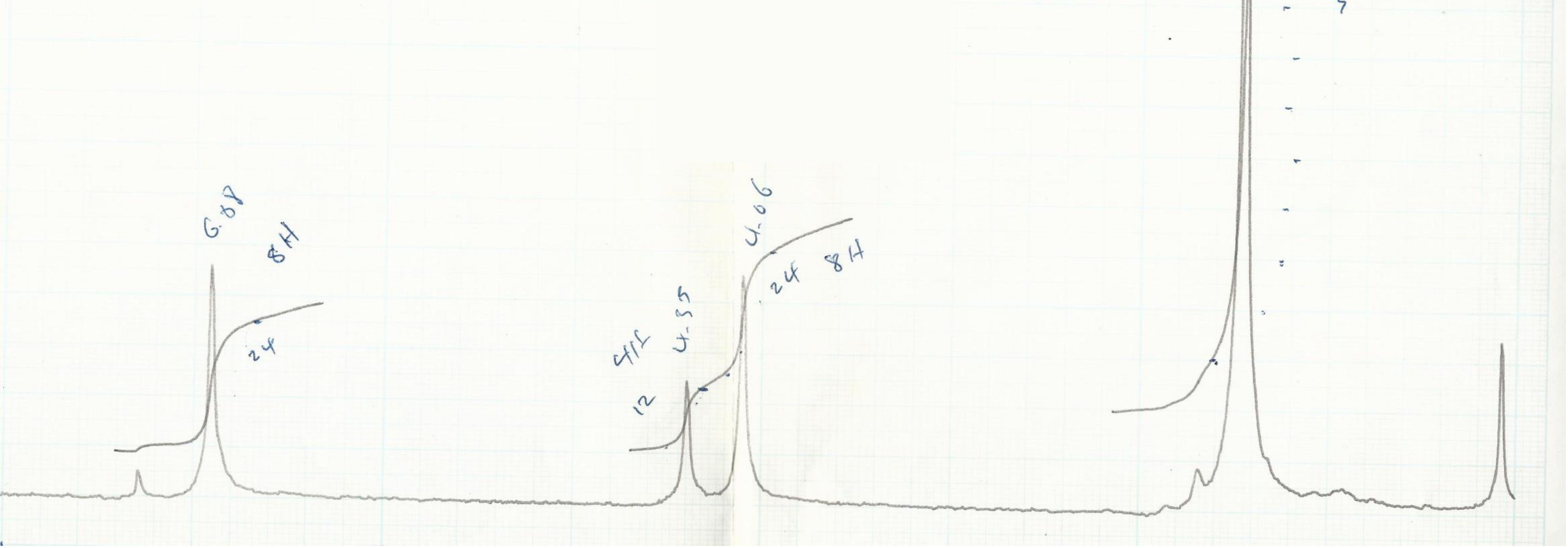

Figure S17 Compound 18a (90 MHz, CDCl3)

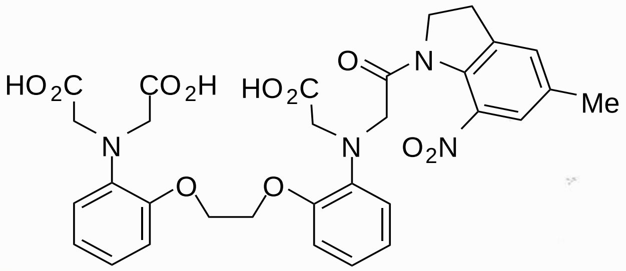

19

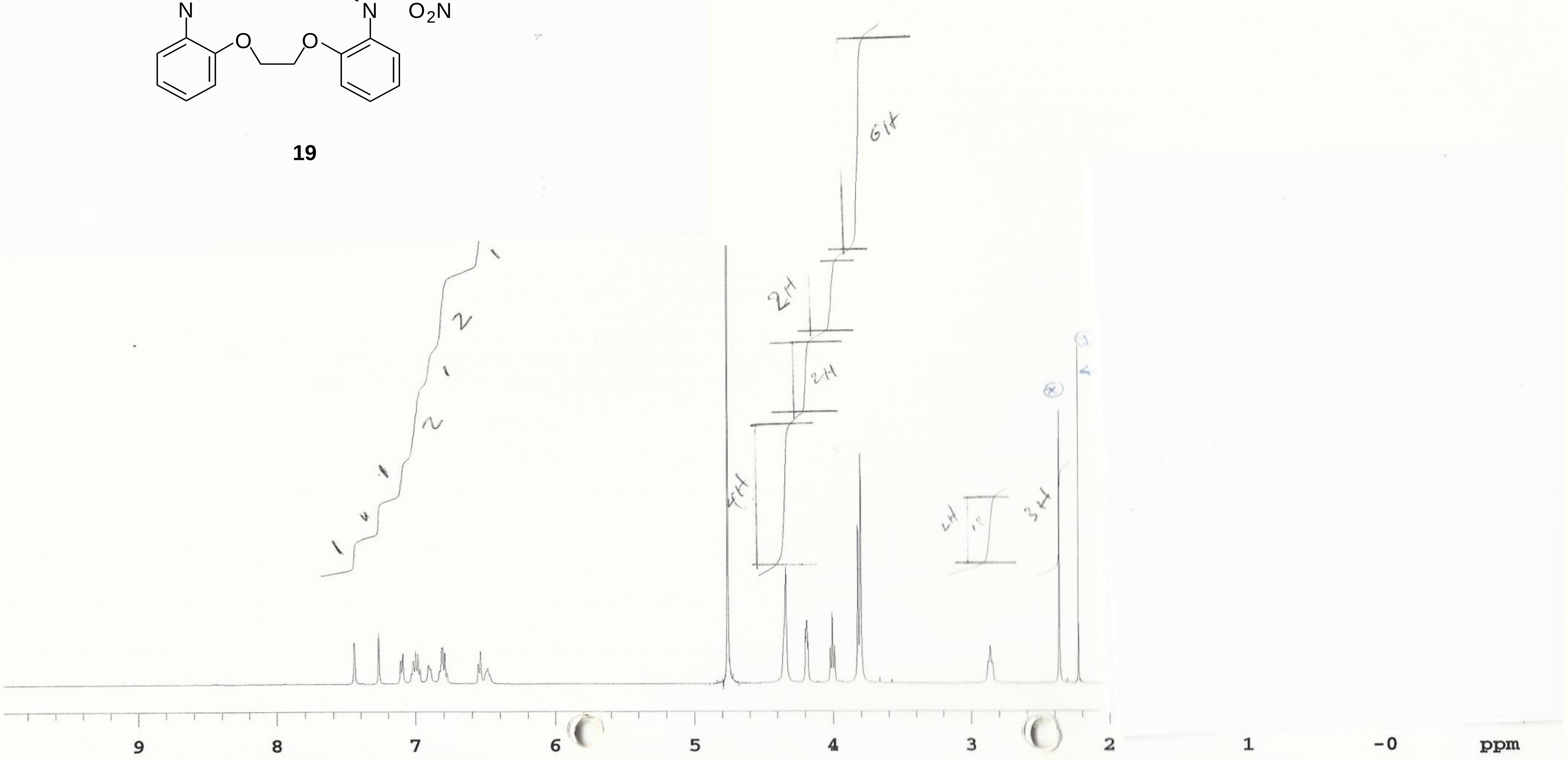

Figure S18 Compound 19 (500 MHz, D2O)

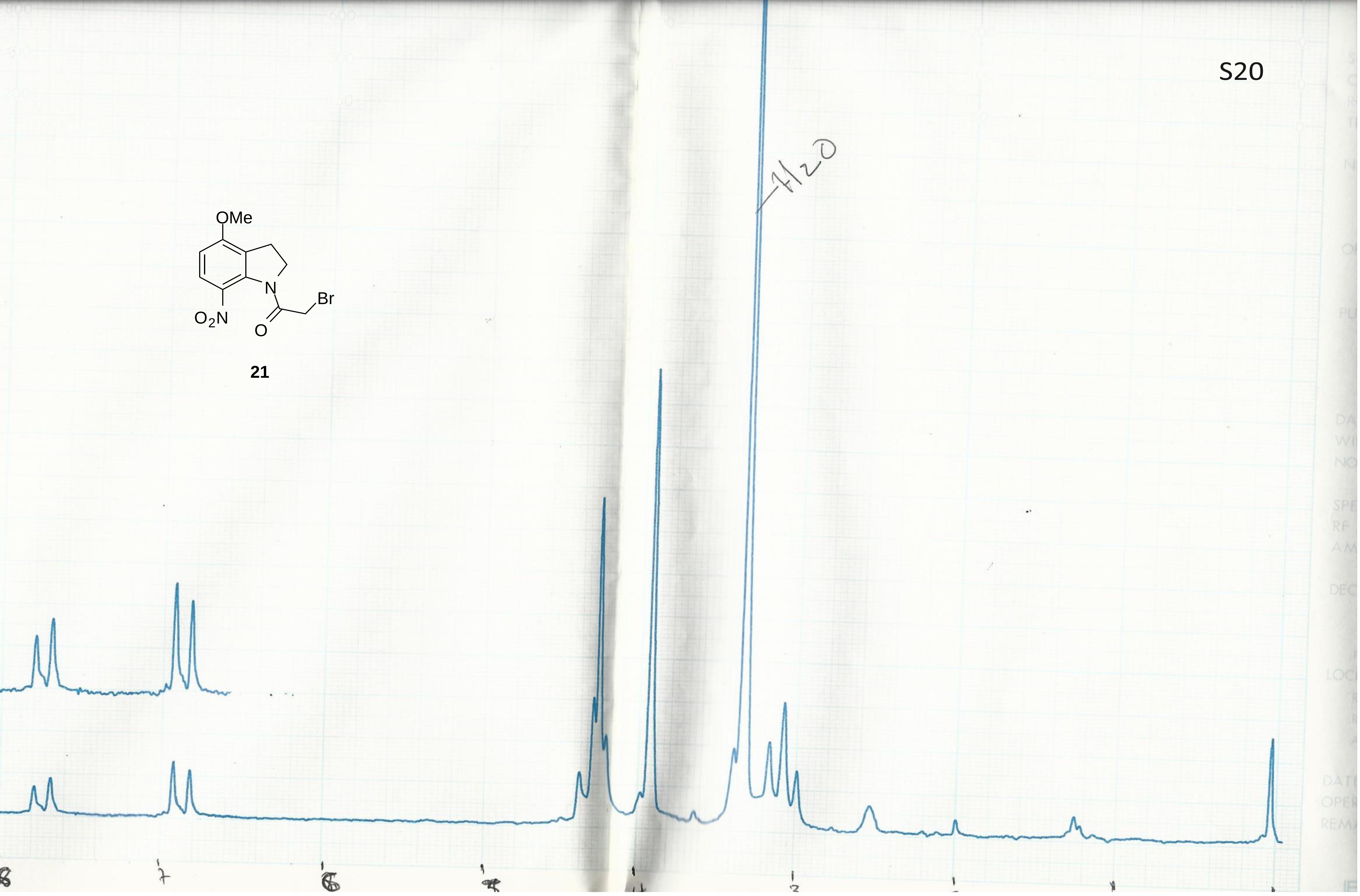

21

Figure S19 Compound 21 (90 MHz, DMSOd6)

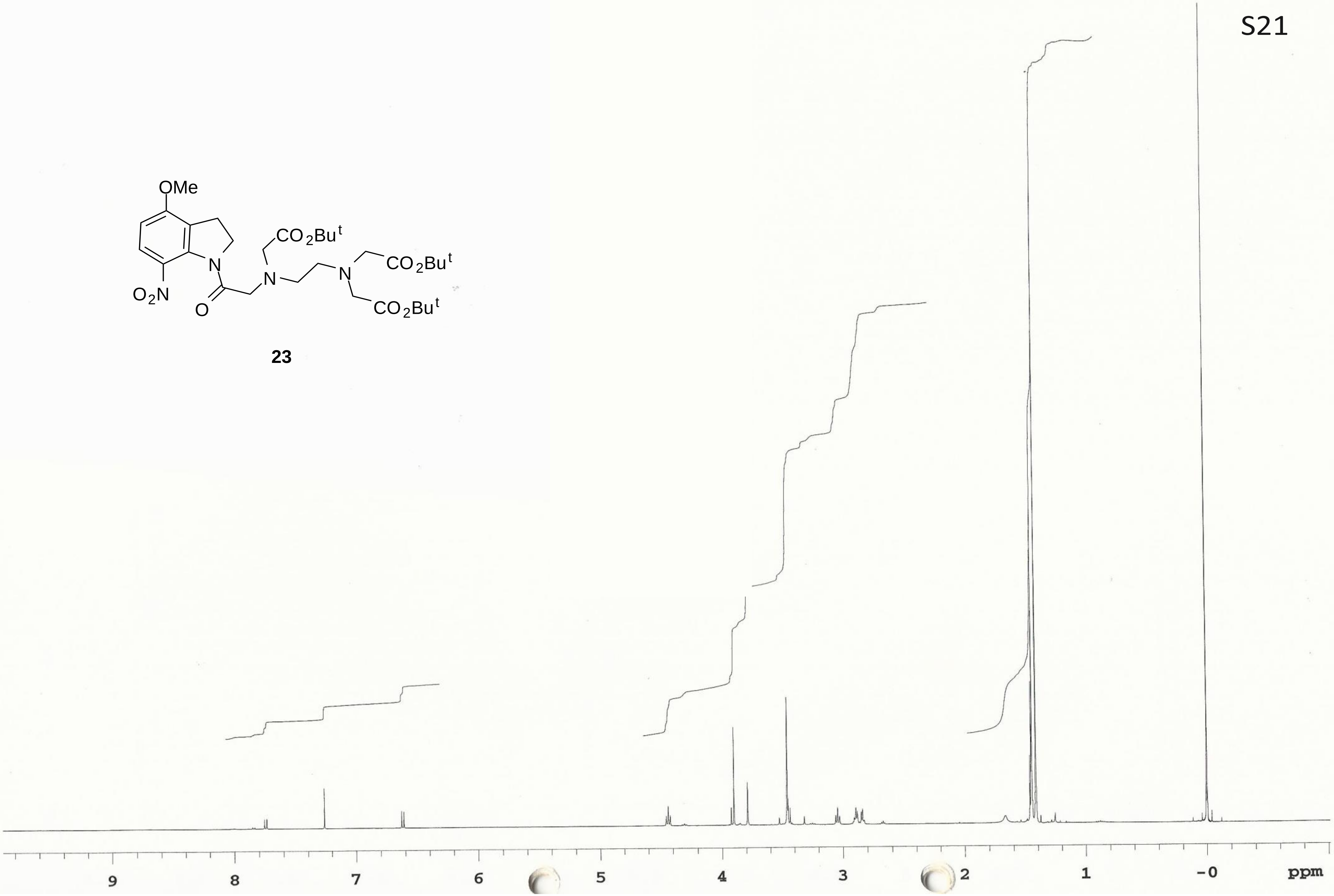

23

Figure S20 Compound 23 (500 MHz, CDCl3)

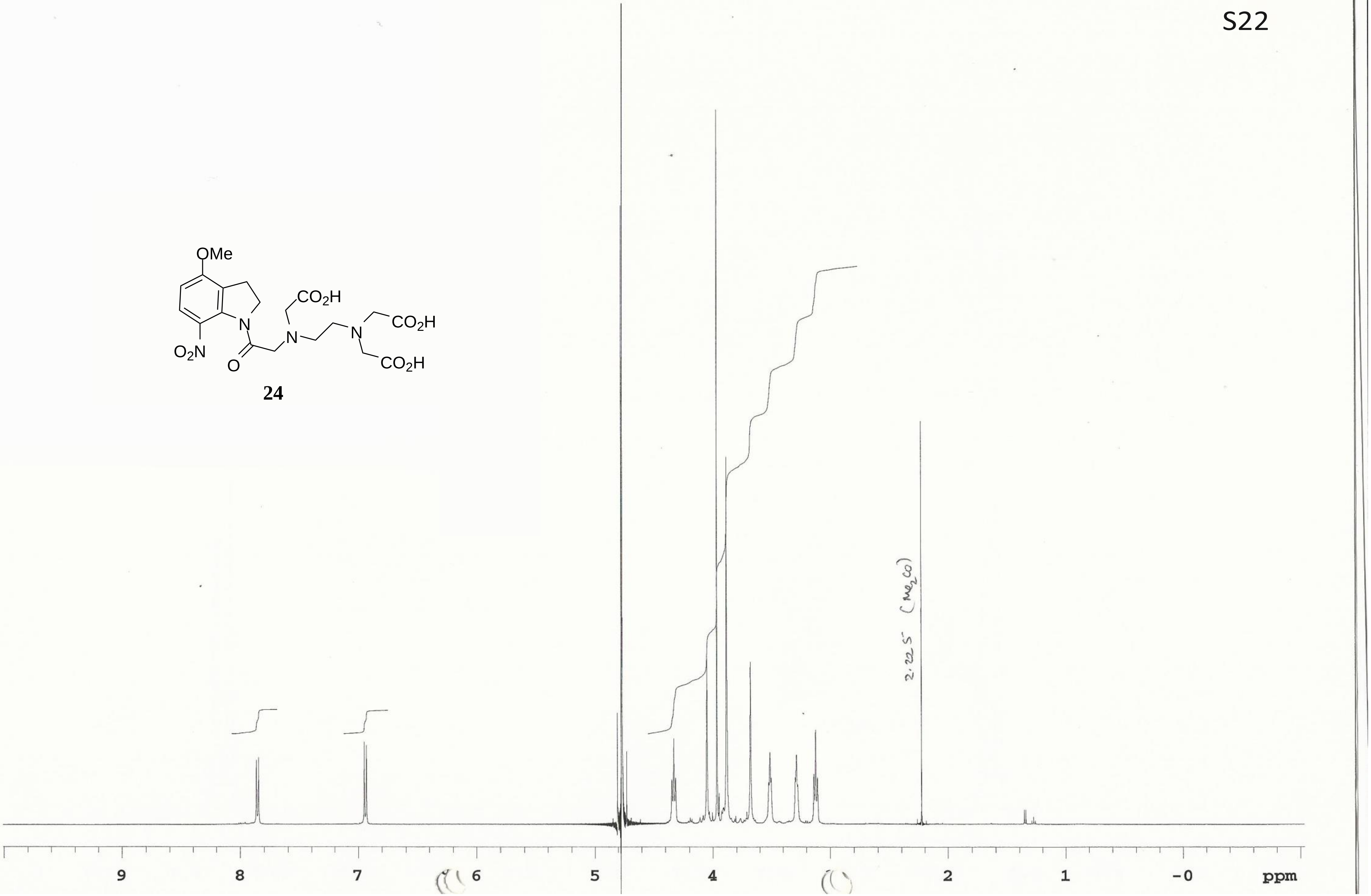

24

Figure S21 Compound 24 (500 MHz, D2O)
